# Challenges in Diagnosis of Pseudo Vasa Previa

**DOI:** 10.1155/2014/903920

**Published:** 2014-05-26

**Authors:** Etsuko Kajimoto, Shinya Matsuzaki, Satoko Matsuzaki, Yusuke Tanaka, Yukiko Kinugasa-Taniguchi, Kazuya Mimura, Takeshi Kanagawa, Tadashi Kimura

**Affiliations:** Department of Obstetrics and Gynecology, Graduate School of Medicine, Osaka University, 2-2 Yamadaoka, Suita, Osaka 565-0871, Japan

## Abstract

Vasa previa is a rare but clinically important obstetrical complication that can be associated with a low-lying placenta or placenta previa. We aim to convey the challenges in diagnosing this condition by presenting 2 cases of pseudo vasa previa diagnosed antenatally as vasa previa using standard and color Doppler ultrasonography. Both patients were falsely diagnosed; only a low-lying placenta was revealed after delivery. These reports emphasize that accurate identification of vasa previa on cervical imaging is important for determining an appropriate treatment strategy.

## 1. Introduction

Vasa previa is a rare but clinically important obstetrical complication in which fetal blood vessels are positioned between the presenting part and cervix, and it can be associated with a low-lying placenta or placenta previa. The estimated incidence of vasa previa is approximately 1 in 2500 deliveries, but it is much higher (1 in 700) among patients who conceive through assisted reproductive technologies. The importance and clinical impact of an antenatal diagnosis of vasa previa is very significant because of the likelihood of adverse fetal outcomes [1]. Because the vessels are attached to the chorion, rupture of fetal membranes can result in fetal bleeding and death within minutes. When the condition is not diagnosed antenatally, the perinatal mortality rate is reported to be approximately 44%, whereas 97% of fetuses survive when an antenatal diagnosis is made, indicating significantly different outcomes [2, 3]. Furthermore, a low-lying placenta is reportedly a risk factor for vasa previa because it occurs in 5% of patients [4]. Accurate diagnosis of vasa previa is therefore crucial. Here, we describe 2 cases that exhibited a low-lying bilobate placenta on transvaginal ultrasonography. Because membranous fetal vessels were observed to pass through the cervical internal os, we diagnosed the patients with vasa previa and administered identical treatments. However, at delivery, it was discovered that both patients had pseudo vasa previa. To the best of our knowledge, no previous study has reported on the clinical features or imaging findings of pseudo vasa previa.

## 2. Case Presentation

### 2.1. Case 1

**Figure 1:**
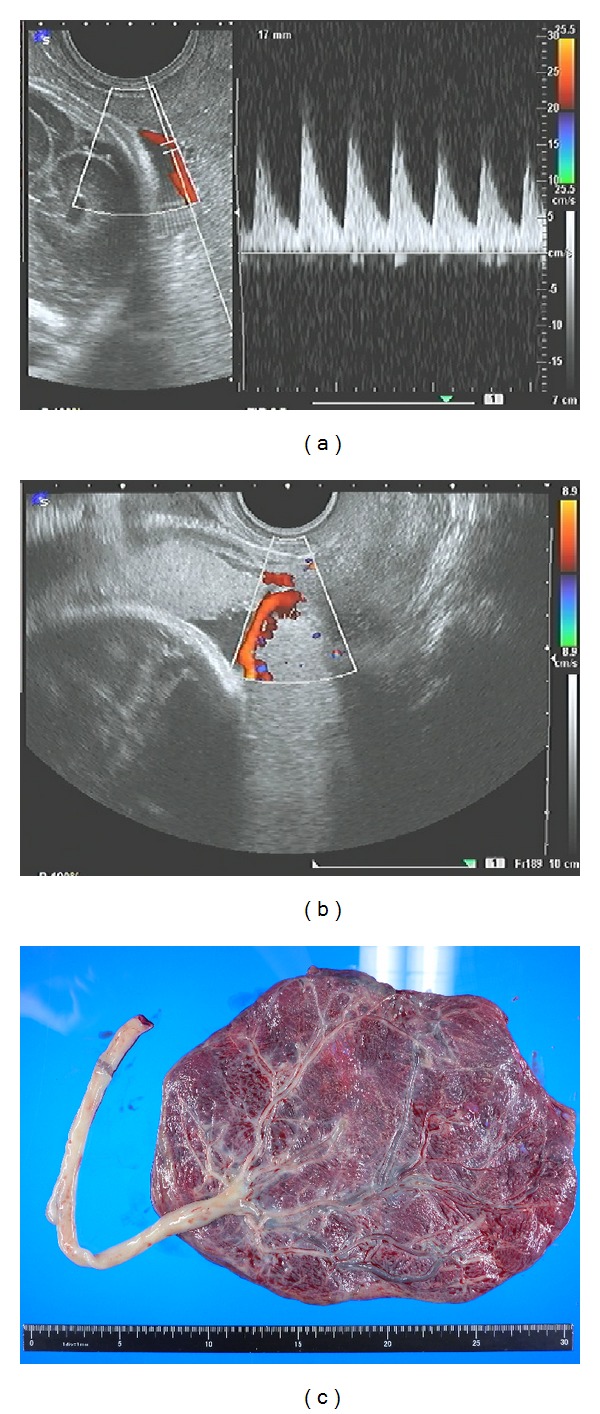
(a and b) First medical examination of the patient at our hospital. Transvaginal ultrasonography revealed membranous fetal vessels passing across the cervical internal os. The findings of color Doppler ultrasonography suggested that the vessels were the umbilical artery. (c) Images of the placenta. Observation during surgery refuted the previous diagnosis of vasa previa.

A 36-year-old female (gravida 1, para 0) with a low-lying placenta was referred to our hospital from a private clinic at 27 weeks of gestation. Transvaginal ultrasonography was performed; the placenta was located near the internal cervical os and was positioned in the posterior to anterior direction. This was recognized as a bilobate placenta. Therefore, the patient was diagnosed with a low-lying bilobate placenta. Membranous fetal vessels were observed to pass across the cervical internal os on real-time color Doppler ultrasonography (Figures 1(a) and 1(b)). The site of cord insertion was unclear because the descending fetal head blocked the view of the placental attachment; therefore, a diagnosis of low-lying placenta complicated by vasa previa was made. The patient was admitted to our hospital at 32 weeks of gestation according to the guidelines for the management of vasa previa by the Society of Obstetricians and Gynecologists of Canada (SOGC) [1]. Fetal growth was found to be appropriate for gestational age.

At 33 weeks of gestation, a nonstress test revealed repetitive variable decelerations; thus, a decision to proceed with the delivery was made. A healthy male infant weighing 1922 g was successfully delivered via cesarean section. The total blood loss during delivery was approximately 1380 mL. Observations made during delivery and evaluation of the placenta refuted our previous diagnosis of vasa previa and bilobate placenta (Figure 1(c)). The postoperative course of the patient was uncomplicated except for the development of endometritis, and she was discharged 10 days after delivery in a healthy condition. The infant was discharged 28 days after delivery in a healthy condition.

### 2.2. Case 2

**Figure 2:**
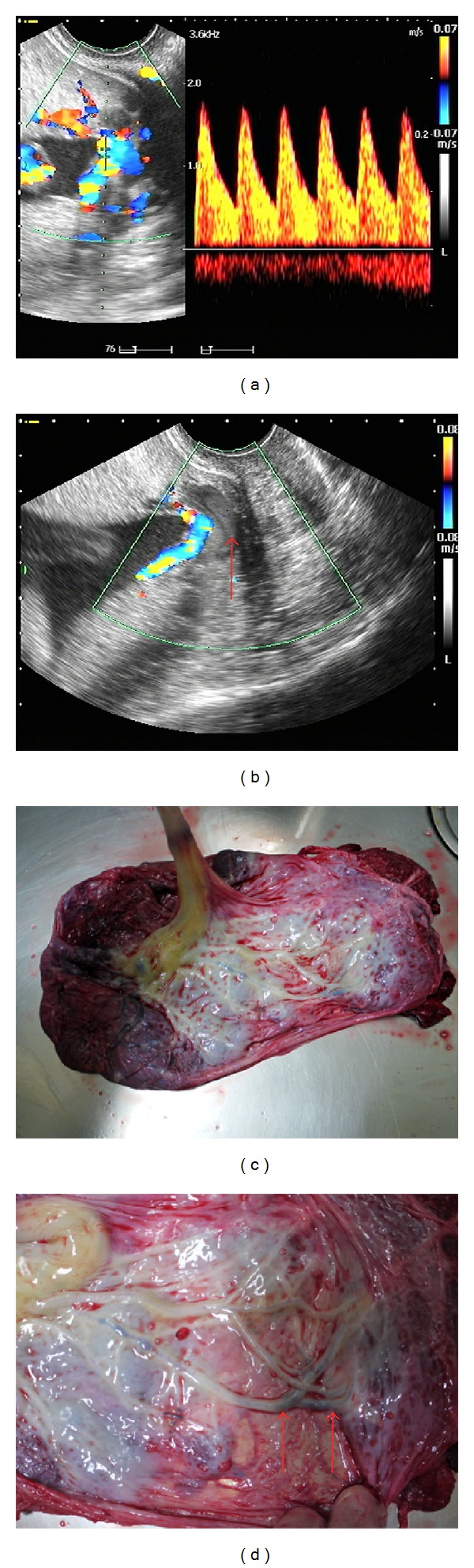
(a and b) Transvaginal ultrasonography revealed membranous fetal vessels passing across the cervical internal os considered to be related to a low-lying bilobate placenta. Therefore, a diagnosis of vasa previa was made. The red arrow indicates a small space between the placental vessels and the internal os. (c and d) Images of the placenta obtained by transvaginal ultrasonography. The red arrows indicate the vessels thought to be vasa previa.

A 38-year-old female (gravida 3, para 0) with increased nuchal translucency (5.9 mm at 13 weeks of gestation) was referred to our hospital from a private clinic at 14 weeks of gestation. Amniocentesis revealed a normal fetal karyotype, while ultrasonography at 19 weeks of gestation revealed a bilobate placenta and no cardiac anomaly. At 27 weeks of gestation, a low-lying bilobate placenta was diagnosed by transvaginal ultrasonography. Furthermore, membranous fetal vessels were observed to pass across the cervical internal os using real-time color Doppler ultrasonography (Figures 2(a) and 2(b)). Therefore, a diagnosis of a low-lying placenta complicated by vasa previa was made.

The patient was admitted to our hospital at 30 weeks of gestation. Fetal growth was found to be appropriate for gestational age. At 32 weeks of gestation, the membranes ruptured and necessitated emergency delivery via cesarean section. A healthy male infant weighing 1708 g was successfully delivered. The total blood loss during delivery was approximately 2400 mL, and the patient received 480 mL of red blood cells by transfusion. Observations made during delivery and evaluation of the placenta refuted the previous diagnosis of vasa previa (Figures 2(c) and 2(d)). The postoperative course of the patient was uncomplicated, and she was discharged 8 days after delivery in a healthy condition. The infant was discharged 50 days after delivery in a healthy condition.

## 3. Discussion

Previously, vasa previa was usually detected by palpation of the fetal vessels within the membranes during labor or on the basis of acute-onset vaginal bleeding and subsequent fetal bradycardia and/or death after membrane rupture. As discussed above, the importance of an accurate diagnosis of vasa previa is significant; if not diagnosed antenatally, the neonatal survival rate is only 44% with a neonatal transfusion rate of 58.5% [2]. A universal screening method for the detection of vasa previa has not yet been established [5] although high-risk factors have been identified [6]. Baulies et al. reported that the incidence of vasa previa was 0.07%, and multivariate analysis revealed the following associated factors in their study. In vitro fertilization (IVF) pregnancies, bilobate or succenturiate placenta, and second trimester placenta previa have been associated with odds ratios of 7.75, 22.11, and 22.86, respectively [6]. Therefore, if patients present with any of these risk factors, a concerted effort to detect vasa previa using ultrasound screening in the second trimester is necessary [7]. Screening for high-risk patients (such as those with IVF pregnancies, a velamentous cord, a low-lying placenta, low cord insertions in the uterus, or a low-lying bilobate placenta) has shown some success [1, 8, 9]. The primary methods of diagnosis are transvaginal ultrasonography and real-time color Doppler ultrasonography, and most cases are diagnosed antenatally.

Case 1 exhibited a placenta located near the internal cervical os and positioned in the posterior to anterior direction. This was recognized as a bilobate placenta, and the surface of the vessels at the placental edge was diagnosed as vasa previa. To achieve an accurate diagnosis of bilobate placenta, ultrasonography should be performed in the second trimester. Unfortunately, in case 1, detailed ultrasonography in the second trimester for the screening of vasa previa was not performed. When the patient was referred to our hospital, the descending fetal head blocked the placental view; therefore, it was difficult to make an accurate diagnosis of bilobate placenta. To avoid a false diagnosis in case 1, light pushing on the cervix to push up the fetal head with a transvaginal probe was performed, and a second scan was performed after a while to achieve an accurate diagnosis. As in case 1, if a patient is diagnosed with a low-lying placenta or placenta previa in the second trimester, detailed ultrasonography should be performed to detect the cord insertion and to rule out bilobate or succenturiate placenta for the accurate diagnosis of vasa previa. Case 2 exhibited a bilobate placenta, and membranous fetal vessels passed across the cervical internal os. The vessels were diagnosed as vasa previa although we ultimately discovered that this was not the case (indicated by red arrows in Figure 2(d)). The red arrow in Figure 2(b) shows the space between the placental vessels and the internal os, indicating placental parenchyma. To avoid a false diagnosis in case 2, serial ultrasonography was performed to identify a space between the placental vessels and internal os to increase the likelihood of an accurate diagnosis of vasa previa. If the obstetrician detects a space between the placental vessels and internal os in low-lying bilobate placenta, careful observation might be needed to rule out pseudo vasa previa. However, in actuality, because of possible adverse fetal outcomes due to vasa previa, case 2 was not difficult to treat as vasa previa.

## 4. Conclusion

We presented 2 cases of pseudo vasa previa. Patients with conditions such as a low-lying bilobate placenta are considered to be at a high risk of vasa previa. If patients with a bilobate placenta are primarily diagnosed with possible vasa previa, accurate identification and exclusion of pseudo vasa previa using transvaginal ultrasound are essential to ensure appropriate and timely treatment.
